# Non-uniform interval-pulse X-ray photon correlation spectroscopy for reduced exposure

**DOI:** 10.1107/S160057752600038X

**Published:** 2026-02-18

**Authors:** Taiki Hoshino, Jingmin Tang

**Affiliations:** a International Center for Synchrotron Radiation Innovation Smart (SRIS) Tohoku University 468-1 Aramaki Aza-Aoba, Aoba-ku Sendai980-8572 Japan; b Institute of Multidisciplinary Research for Advanced Materials Tohoku University 2-1-1 Katahira, Aoba-ku Sendai980-8577 Japan; European XFEL, Germany

**Keywords:** X-ray photon correlation spectroscopy, X-ray speckle visibility spectroscopy, X-ray radiation damage

## Abstract

X-ray photon correlation spectroscopy (XPCS) is a powerful technique for evaluating microscopic dynamics using coherent X-rays. Detecting fast or small-scale dynamics typically requires strong illumination and wide-angle scattering detection; however, such conditions can cause non-negligible sample damage. This study presents a non-uniform pulse-interval XPCS approach that enables quantitative dynamical analysis with substantially reduced X-ray exposure. In conventional XPCS, continuous acquisition at uniform time intervals leads to long cumulative exposure, which can introduce radiation-induced artefacts. In this study, only 11 scattering images were recorded at non-uniform intervals, providing a broad range of delay times from a single measurement and enabling dense temporal sampling without increasing the exposure dose. The resulting dataset was analyzed using both XPCS- and X-ray speckle visibility spectroscopy (XSVS)-based schemes, and the results demonstrated that these two independent analyses yield consistent relaxation behaviors. The proposed approach offers an efficient framework for probing complex or non-Brownian dynamics in radiation-sensitive materials and expands the applicability of XPCS to soft and biological systems.

## Abstract 2

In this work, non-uniform interval-pulse X-ray photon correlation spectroscopy enabled the measurement of dynamics efficiently while reducing X-ray exposure by two orders of magnitude. This approach facilitates dense delay-time sampling and minimizes radiation damage, offering a powerful tool for studying sensitive systems.

## 1. Introduction

X-ray photon correlation spectroscopy (XPCS) is used to evaluate microscopic dynamics by illuminating a sample with partially coherent X-rays and analyzing temporal fluctuations in the resulting scattering patterns. In conventional XPCS, coherent X-ray speckle images are repeatedly acquired at fixed exposure times and uniform time intervals, and various correlation functions are computed to characterize the dynamics of the scatterers (Sutton, 2008 ▸). X-ray induced changes in dynamics have been observed for samples that are sensitive to X-ray irradiation (Ruta *et al.*, 2017 ▸; Chushkin *et al.*, 2022 ▸; Timmermann *et al.*, 2023 ▸). As synchrotron radiation sources are being upgraded to fourth-generation facilities worldwide and continue to provide increased brightness, obtaining reliable dynamical information while avoiding irradiation effects has become increasingly important. The development of high-speed detectors has facilitated sub-microsecond measurements using storage rings (Zhang *et al.*, 2018 ▸; Jo *et al.*, 2021 ▸; Chushkin *et al.*, 2025 ▸). Additionally, XPCS experiments using pulsed X-ray free-electron laser (XFEL) sources have been reported (Carnis *et al.*, 2014 ▸; Lehmkühler *et al.*, 2015 ▸, 2018 ▸), and, at the European XFEL, measurements of megahertz-scale dynamics using megahertz-pulse trains are now possible (Lehmkühler *et al.*, 2020 ▸; Reiser *et al.*, 2022 ▸; Girelli *et al.*, 2025 ▸; Jo *et al.*, 2025 ▸). Various experimental strategies are being developed to take full advantage of these rapidly evolving light sources, while mitigating radiation damage and overcoming limitations imposed by detector frame rates and photon statistics.

X-ray speckle visibility spectroscopy (XSVS) has been developed as an extension of XPCS. This technique was originally introduced in visible-light laser experiments (Dixon & Durian, 2003 ▸; Bandyopadhyay *et al.*, 2005 ▸); more recently, several implementations using X-rays have been reported (Inoue *et al.*, 2012 ▸; DeCaro *et al.*, 2013 ▸; Li *et al.*, 2014 ▸; Sun *et al.*, 2020 ▸). XSVS can be broadly categorized into two categories. The first approach investigates the dependence of speckle visibility on the exposure time. In this method, scattering images are recorded with various exposure times within a single-frame acquisition and the dynamics are inferred from the rate at which the visibility decreases. Faster scatterer motion leads to more rapid speckle fluctuations, resulting in a quicker decay in visibility. By controlling the analyzed exposure time, this approach can probe time scales beyond the frame-rate limitation of the detector. It also enables dynamical analysis under very low dose conditions where the scattered intensity is extremely weak (Verwohlt *et al.*, 2018 ▸; Möller *et al.*, 2021 ▸). However, accessing long relaxation times requires long-exposure data, and, for systems exhibiting slow dynamics, the X-ray intensity must be significantly reduced to avoid radiation damage during these extended exposures.

The second approach overlays two frames recorded with different time separations and evaluates the resulting reduction in visibility, a method known as double-pulse XSVS (DP-XSVS) (Gutt *et al.*, 2009 ▸). By varying the inter-frame delay and shifting the beam position for each measurement, the irradiation time at any given position is limited to two frames, thereby greatly reducing radiation damage. Owing to this two-frame requirement, DP-XSVS has been effectively combined with XFEL pulse sources and optical delay units (Roseker *et al.*, 2018 ▸; Shinohara *et al.*, 2020 ▸; Dallari *et al.*, 2021 ▸; Sun *et al.*, 2021 ▸; Majumdar *et al.*, 2024 ▸). This approach makes it possible to achieve primary target time scales in the femtosecond to nanosecond range.

In both approaches, measurements must be performed at multiple delay times and the sample position must be shifted after each measurement to avoid cumulative irradiation. Consequently, the total measurement time can become extremely long. Depending on the number of exposure-time patterns and sample positions, 10^2^–10^4^ individual measurements may be required. With high-intensity X-rays, such as those from XFELs, the beam position must be changed frequently after every two frames to prevent damage; however, particularly in synchrotron storage-ring experiments, it is generally feasible to obtain several frames at a single position without significant degradation, and recent advances indicate that this is also achievable at XFELs under optimized irradiation conditions.

In practice, an optimal experimental strategy must balance irradiation damage, the required time scales, the accessible *q* range (where *q* denotes the magnitude of the scattering vector) and the total available measurement time. For simple Brownian motion, the temporal autocorrelation function is a single exponential; therefore, a relatively small number of delay times may be sufficient. However, many systems studied using XPCS are more complex than simple liquids and their autocorrelation functions often deviate from a single-exponential form; consequently, evaluating these deviations is essential. Therefore, it is desirable to acquire a sufficiently large number of delay-time data points.

In this study, we demonstrate a practical approach for efficiently obtaining temporal autocorrelation functions while mitigating radiation damage. This approach is applied to a polymer gel that exhibits moderate sensitivity to X-ray exposure. Unlike standard XSVS, which evaluates only two frames, multiple speckle images – typically up to ∼10 frames – are acquired at non-uniform time intervals at a single beam position in the proposed approach. This significantly increases the number of available delay-time combinations, enabling a substantial enhancement in the density of delay-time sampling and allowing XSVS measurements to be performed more efficiently. Recent advances in detector technology have significantly improved the accessible time resolution of serial XPCS. The present approach is applicable to both XPCS- and XSVS-based analyses. In this study, we perform and compare dynamical analyses based on both XPCS and XSVS using the same non-uniform pulse dataset.

## 2. Methods

### 2.1. Conventional XPCS

In conventional XPCS, a sequence of *N* coherent X-ray images is recorded at uniform time intervals, and the temporal intensity autocorrelation function is calculated as follows (Sutton, 2008 ▸),
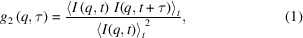
where 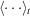 denotes averaging time and τ relates to delay times. When temporal evolution is important, the two-time correlation function is evaluated instead (Brown *et al.*, 1997 ▸; Malik *et al.*, 1998 ▸),
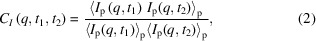
where 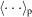 denotes averaging over all detector pixels within a *q* ring of width *dq*. For systems in equilibrium, the conventional correlation function *g*_2_ can be obtained by time-averaging the two-time correlation function as follows,
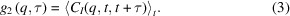


### 2.2. XPCS using log pulses

We next consider XPCS measurements performed using a sequence of non-uniform pulse intervals. Let us assume that *N* (>2) images are obtained from a single measurement. By introducing non-uniform time spacing between frames, a single measurement provides *N*(*N* − 1)/2 distinct delay times τ. Using equation (3), these delay times yield a *g*_2_ function containing *N*(*N* − 1)/2 data points. In practice, however, log-pulse schemes are designed such that no duplicate delay-time pairs are produced. Consequently, the time-averaging 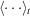 in equation (3) is not performed. Instead, each individual two-time correlation value *C*_*I*_(*q*, *t*, *t* + τ) is directly used as the corresponding *g*_2_(*q*, τ) value.

### 2.3. XSVS using log pulses

In DP-XSVS, the scattering intensities *I*(*t*) and *I*(*t* + τ) obtained from two frames separated by a delay time τ are considered. If the speckle contrast from a single frame is denoted by β_SF_(*q*), the speckle contrast β_DF_(*q*, τ) of the summed image formed from the two frames is given by (Gutt *et al.*, 2009 ▸)
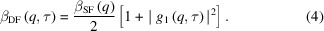
Here, 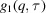 is the intermediate scattering function, which is related to 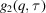 via the Siegert relation, 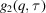 = 1 + 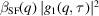. The probability distribution function *P*(*K*) for observing *K* photon counts in a speckle pattern can be expressed as follows (Mandel, 1959 ▸; Goodman, 1985 ▸),
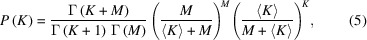
where 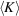 is the mean count rate, Γ is the relaxation rate and *M* is the number of modes.

In conventional DP-XSVS, a pair of frames with a delay τ is acquired in each measurement and data are collected for various values of τ. In the present study, we considered acquiring *N* (>2) scattering frames in a single measurement, referred to as multi-pulse XSVS. By introducing non-uniform time spacing between frames, a single measurement yields *N*(*N* − 1)/2 distinct delay times τ. For each τ, the speckle contrast β_DF_ = 1/*M* can be obtained using equation (4), providing information equivalent to that of the temporal autocorrelation function.

### 2.4. Experiments

XPCS and XSVS measurements were performed using beamline BL10U at NanoTerasu. NanoTerasu is a 3 GeV high-brilliance synchrotron radiation facility (Obara *et al.*, 2025 ▸; Nishimori *et al.*, 2025 ▸) and BL10U is a vacuum-sealed undulator beamline that provides tender to hard X-rays (Ishiguro *et al.*, 2024 ▸). In this study, X-rays with an energy of 12.40 keV were selected using a Si (111) double-crystal monochromator. During the experiments, the storage-ring current at NanoTerasu was 320 mA.

A partially coherent X-ray beam was defined to 20 µm × 20 µm using slits placed upstream of the sample. The beam illuminated the sample, and the scattered X-rays were detected 9.3 m downstream using an EIGER 1M two-dimensional photon-counting detector (DECTRIS, Switzerland) with a pixel size of 75 µm × 75 µm. The incident flux was attenuated to approximately one third using attenuators. At the sample position, the beam size was ∼30 µm × 30 µm (full width at half-maximum) and the flux was 2 × 10^9^ photons s^−1^.

**Figure 1:**
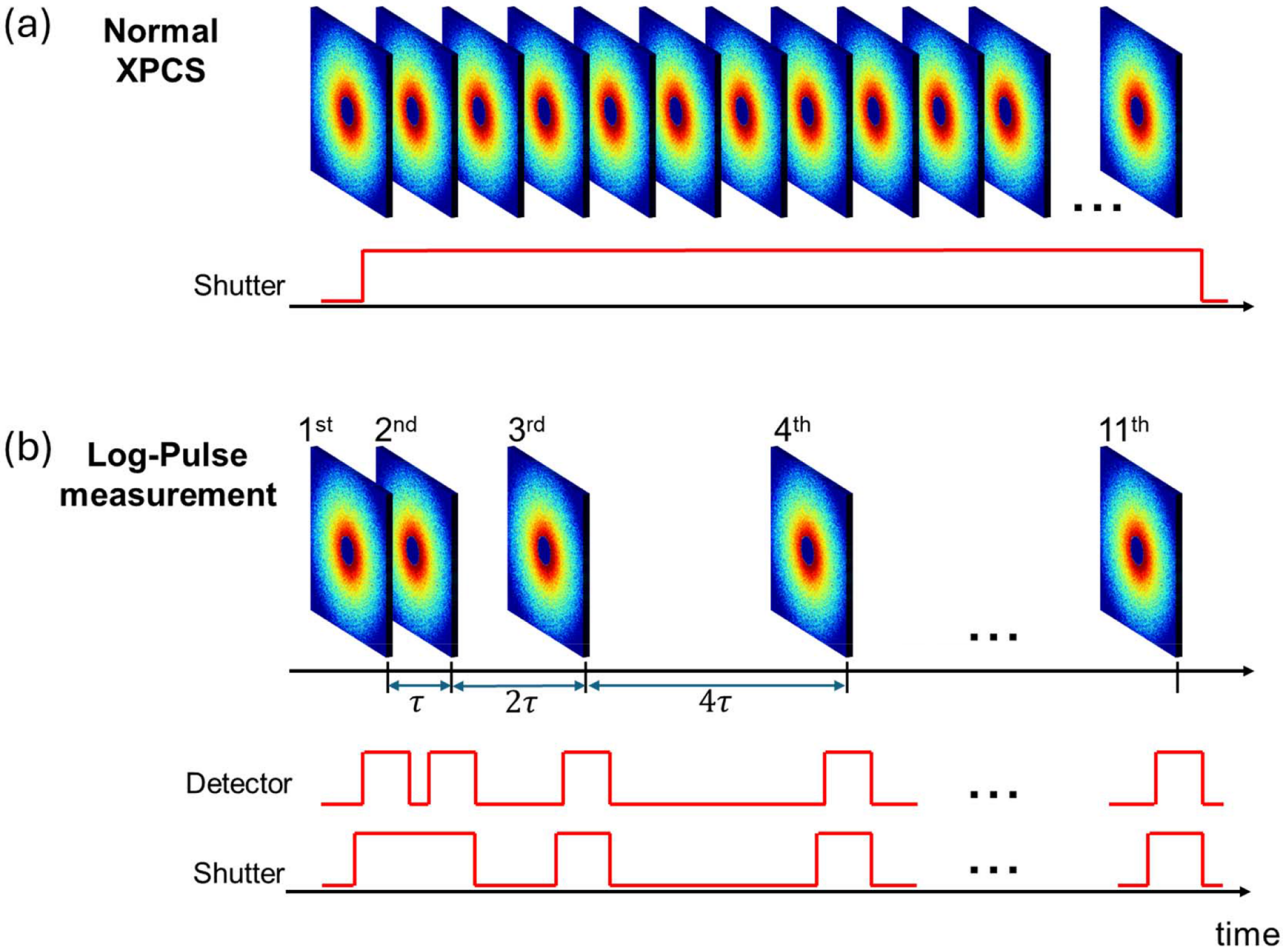
Illustration of two data-acquisition schemes. (*a*) Normal XPCS measurement: 1000 frames were acquired with an exposure time of 80 ms per frame, with the shutter kept open throughout. (*b*) Log-pulse measurement: 11 scattering images with an exposure time of 60 ms were recorded at times *t* = *nt*_f_, with *n* = 1, 2, 4, 8, 16, 32, 64, 128, 256, 512 and 1024, where *t*_f_ = 80 ms. Apart from during exposure periods, the shutter was kept closed.

Two data-acquisition schemes are illustrated in Fig. 1 ▸. For comparison, both conventional continuous time-resolved measurements and log-pulse time-resolved measurements were conducted. In the standard XPCS measurement, 1000 frames were acquired with an exposure time of 80 ms per frame (*t*_f_), while the shutter remained open throughout, resulting in a total exposure time of 80 s. In the log-pulse time-resolved measurements, 11 scattering images with an exposure time of 60 ms were recorded at times *t* = *nt*_f_, with *n* = 1, 2, 4, 8, 16, 32, 64, 128, 256, 512 and 1024, where *t*_f_ = 80 ms. These 11 frames provided 55 distinct delay times τ through pairwise combinations. Pulse control was implemented using the Digital Discovery module (DIGILENT Inc.), which synchronized the shutter and detector. Apart from during exposure periods, the shutter remained closed to prevent sample illumination. To compensate for shutter-opening latency, the trigger signal was configured to rise 30 ms before the intended opening time.

The sample was a polymer gel prepared by mixing poly­(vinyl alcohol), borax (FUJIFILM Wako Pure Chemical Corporation, Japan) and pure water. During gel synthesis, silica particles with a diameter of 120 nm (Nissan Chemicals, Japan) were dispersed at a volume fraction of 1 vol%. The sample was sealed inside a 2 mm-diameter quartz capillary and the top was covered with ep­oxy resin to prevent drying. Measurements were repeated 30 times while shifting the irradiation position by 50 µm for each repetition.

## 3. Results and discussion

### 3.1. Conventional XPCS results

**Figure 2:**
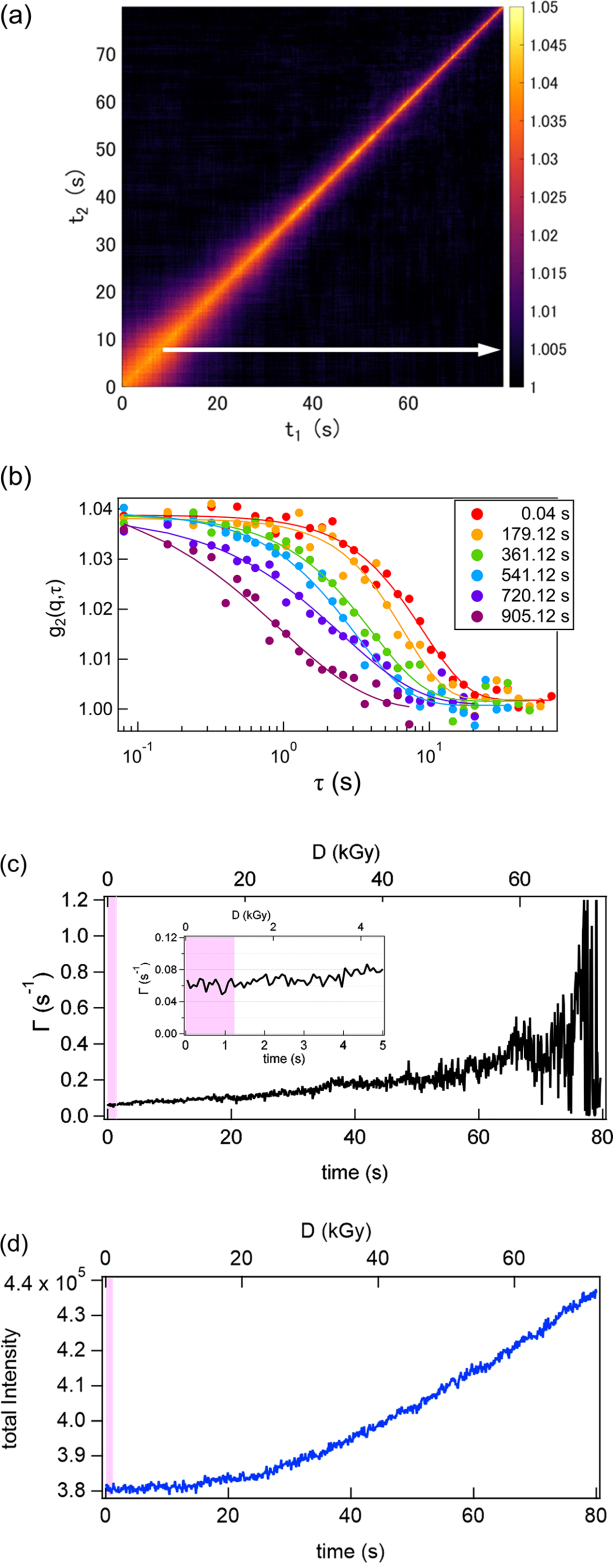
(*a*) Two-time correlation function at *q* = 0.0190 nm^−1^ obtained from the conventional XPCS measurement. (*b*) Time correlation functions extracted at several times by horizontally slicing the two-time correlation function. (*c*) Temporal evolution of Γ obtained from the fitting by equation (6). (*d*) The temporal evolution of the total scattered intensity obtained by summing all pixels used in the analysis. The colored regions in (*c*) and (*d*) indicate the time corresponding to the total exposure duration of the log-pulse measurement.

Fig. 2 ▸ shows the results obtained from conventional XPCS measurements. Fig. 2 ▸(*a*) illustrates the two-time correlation function at *q* = 0.0190 nm^−1^. In this study, the width of the *q* ring *dq* was 0.003 nm^−1^. A clear decrease in the relaxation time was observed as the measurement progressed. Initially, the relaxation time was of the order of several seconds; however, toward the end, it became as fast as the limit of the measurement time window. Fig. 2 ▸(*b*) shows the time correlation functions extracted at several times by horizontally slicing the two-time correlation function, as indicated by the arrow in Fig. 2 ▸(*a*) (Bikondoa, 2017 ▸). All curves were well described by the following function,
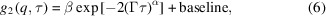
where β is the speckle contrast determined by the experimental conditions, Γ is the relaxation rate and α is the stretched or compressed exponent.

Fig. 2 ▸(*c*) shows the temporal evolution of Γ obtained from the above fitting. The figure also shows the accumulated dose, calculated using the estimated dose rate of 0.9 kGy s^−1^ based on the sample composition. No clear trend is observed during the first 1.5 s (corresponding to a dose of ∼1.4 kGy), but thereafter a gradual increase can be observed. Furthermore, the temporal evolution of the total scattered intensity – obtained by summing all pixels used in the analysis – shown in Fig. 2 ▸(*d*), remains nearly constant during the first 10 s, then increases rapidly. These trends indicate that X-ray irradiation gradually alters the gel structure and accelerates the motion of the probe particles. Under such conditions, the intrinsic dynamics of the system cannot be evaluated without the influence of X-ray irradiation.

### 3.2. Log-pulse XPCS results

**Figure 3:**
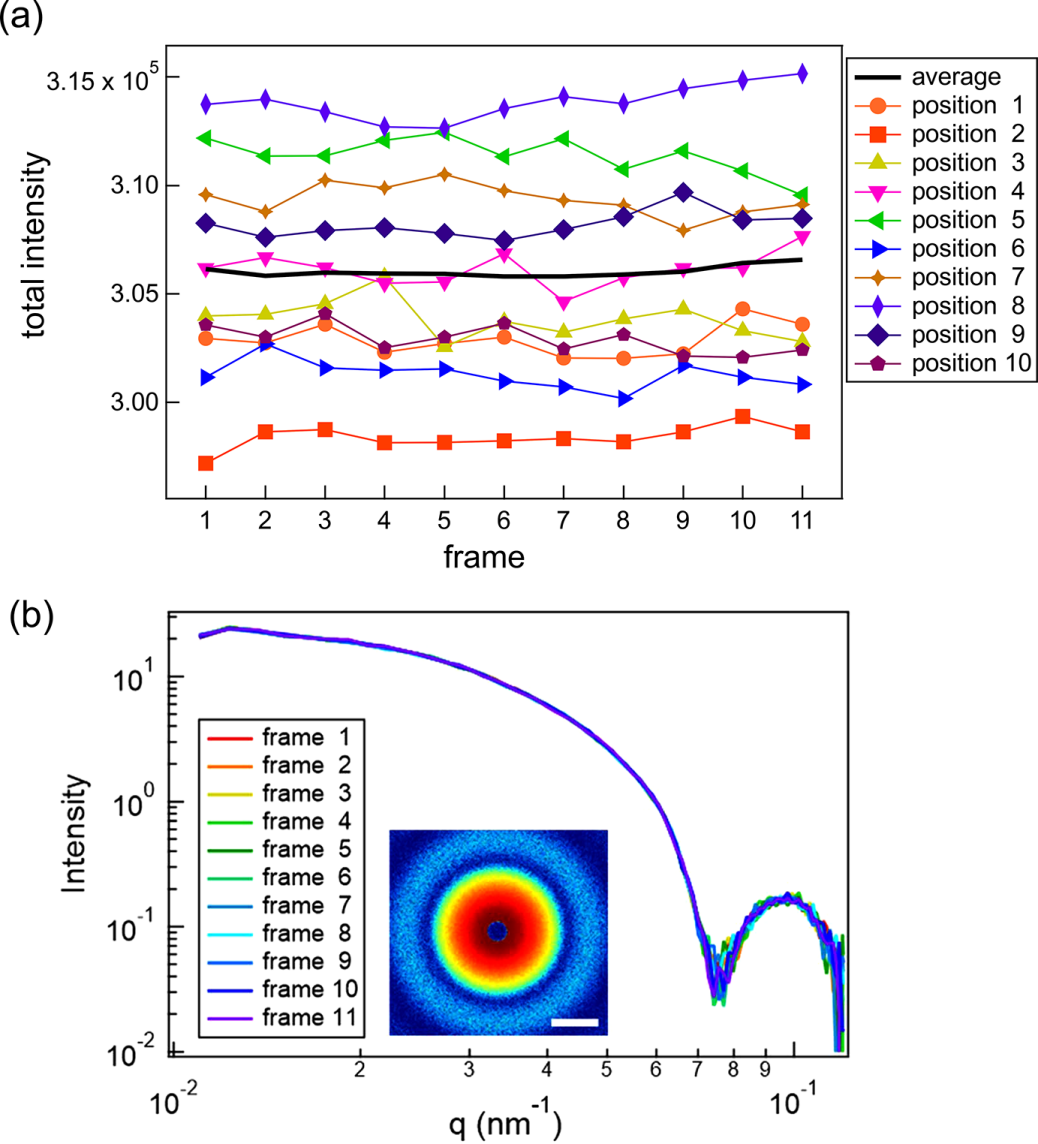
(*a*) Frame dependence of the total scattered intensity obtained from log-pulse time-resolved measurements. (*b*) Representative scattering profiles obtained from 11 frames, with the corresponding summed image. The intensity is displayed on a logarithmic scale and the scale bar corresponds to 0.05 nm^−1^.

Fig. 3 ▸(*a*) shows the frame dependence of the total scattered intensity obtained from the log-pulse time-resolved measurements, plotted for ten representative positions along with the average over all 30 positions. Fig. 3 ▸(*b*) illustrates the representative scattering profiles obtained from 11 frames. The total irradiation time in this measurement, including the shutter opening and closing periods, was less than 1.26 s (11 exposures of 60 ms and shutter operation times of ≤30 ms, repeated 20 times). As highlighted by the colored regions in Figs. 2 ▸(*c*) and 2 ▸(*d*), this duration is shorter than the timescale over which changes in the dynamics or intensity become apparent. In both cases, the values remained nearly constant, indicating that no significant change in scattering intensity occurred over the 11 frames due to X-ray irradiation.

**Figure 4:**
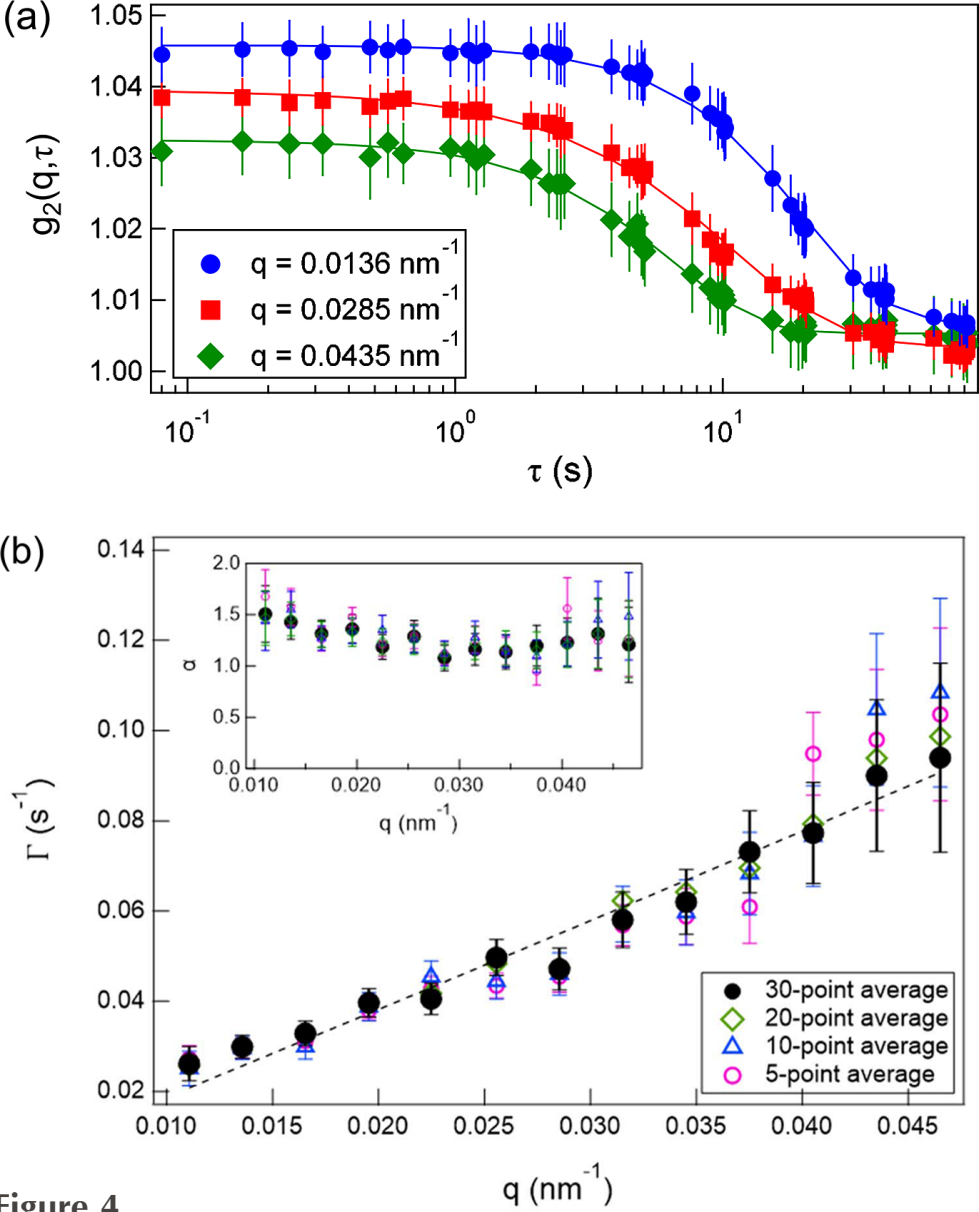
(*a*) Representative *g*_2_(*q*, τ), averaged over 30 positions. (*b*) The *q* dependence of Γ and α (inset) for different numbers of averaged positions. The dashed line represents a power-law fit to the data averaged over 30 positions.

Fig. 4 ▸(*a*) shows the *g*_2_ function (averaged over 30 positions) calculated using equation (3), as described in Section 2.2. The error bars represent the standard deviation of the average over the 30 positions. As expected, larger *q* values exhibited faster relaxation. From the 11 scattering images, 55 delay times τ were obtained and plotted. All curves were well described by equation (6). In this low-*q* range, the contrast is expected to remain nearly constant and independent of *q* based on the calculations considering the optical setup (Hruszkewycz *et al.*, 2012 ▸); however, the speckle contrast β decreased with increasing *q*. As seen in Fig. 3 ▸(*b*), the scattering intensity decreases by approximately one order of magnitude across this *q* range. Across a larger *q* range, the signal-to-background ratio decreases, leading to a reduction in speckle contrast, as reported in previous studies (Lhermitte *et al.*, 2017 ▸; Chèvremont *et al.*, 2024 ▸). Additionally, the baseline is ∼1.004, slightly higher than unity. This can be attributed to very slow dynamical modes present in the gel.

The values of Γ and α obtained from the fitting are shown in Fig. 4 ▸(*b*) for different numbers of averaged positions. Although the *q* dependence was already evident even with relatively few averages, the scatter decreased as the number of averaged positions increased. A power-law fit of the relaxation rate for the 30-point average Γ ∝ *q*^*n*^ yielded *n* ≈ 1, whereas α considered values greater than unity. These features indicate hyperdiffusive motion, which is characteristic of soft solids such as gels, polymer networks, ep­oxy resins, and polymers near the glass-transition temperature (Hoshino *et al.*, 2013 ▸, 2021 ▸; Ruta *et al.*, 2014 ▸; Hernández *et al.*, 2015 ▸; Frenzel *et al.*, 2019 ▸; Yavitt *et al.*, 2021 ▸). Therefore, the motion of the particles dispersed within the gel was clearly captured.

### 3.3. Log-pulse XSVS results

**Figure 5:**
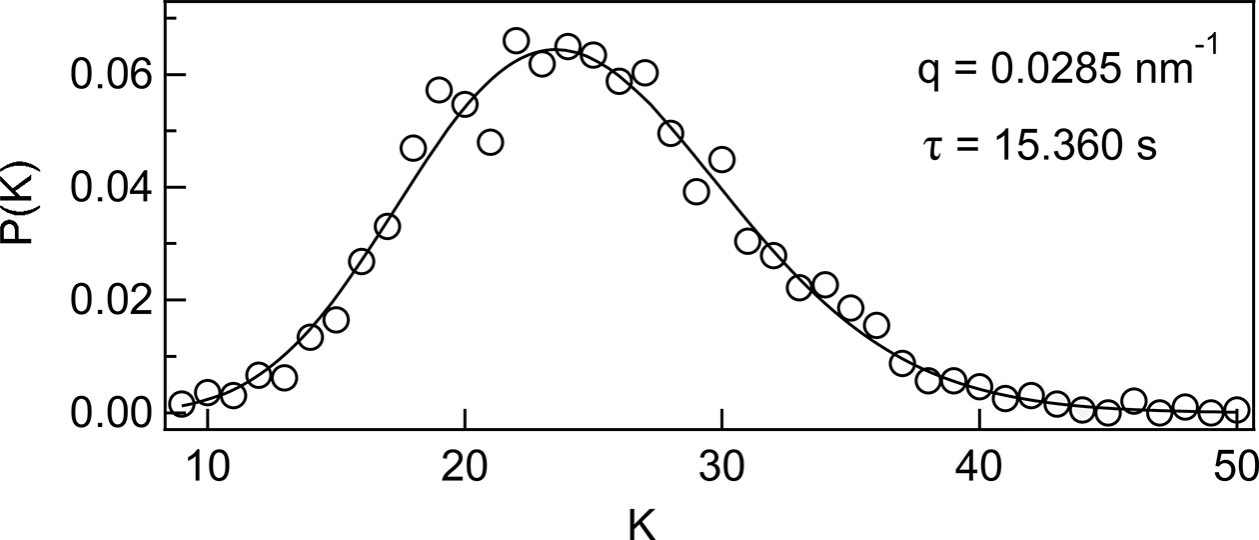
Representative intensity distribution obtained by summing the speckle images from the seventh and eighth frames (τ = 15.360 s) at *q* = 0.0285 nm^−1^ with *dq* = 0.003 nm^−1^.

Fig. 5 ▸ shows a representative intensity distribution obtained by summing the speckle images from the seventh and eighth frames (τ = 15.360 s) at *q* = 0.0285 nm^−1^ with *dq* = 0.003 nm^−1^. When this distribution was fitted using equation (5), with 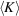 and *M* as free parameters, the fit was in good agreement with the data, as shown in the figure. The same analysis was applied to all 55 combinations of frame pairs generated from the 11 frames.

**Figure 6:**
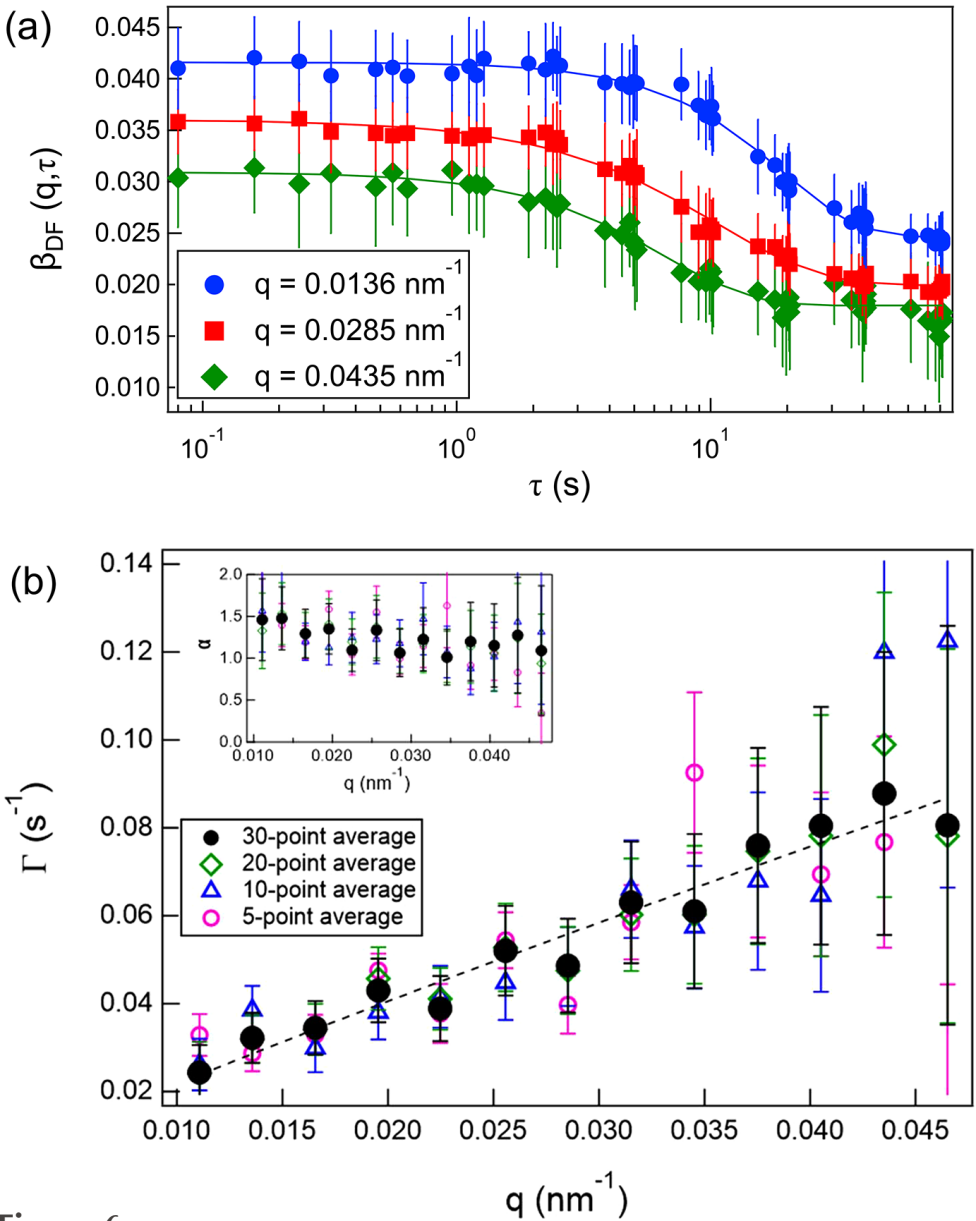
(*a*) Representative β_DF_(*q*, τ), averaged over 30 positions. (*b*) The *q* dependence of Γ and α (inset) for different numbers of averaged positions. The dashed line represents a power-law fit to the data averaged over 30 positions.

The obtained representative β_DF_(*q*, τ), averaged over 30 positions, are plotted in Fig. 6 ▸. The error bars represent the standard deviation of the average over the 30 positions. The solid lines indicate fits to the following function, which has the same form as equation (6),
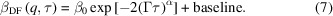
Here, β_0_ is the contrast prefactor corresponding to the speckle contrast at τ → 0. All curves showed good agreement with the fitting function. The *q* dependence of the baseline is more pronounced than the results obtained by log-pulse XPCS. This is because, in XSVS, the contrast contributes not only to the relaxation term but also to the baseline [equation (4)], and the difference in contrast is considered to originate from the ratio of the scattering intensity to the background, as discussed previously. The relaxation rate Γ and the exponent α obtained from these fits are shown in Fig. 6 ▸(*b*) for different numbers of averaged positions. Even for a small number of averaged points, a clear increasing trend with *q* was observed, and the scatter decreased as the number of averages increased. The *q* dependence of the relaxation rate was described by Γ ∝ *q*^0.9^, and α again took values greater than unity. These trends were consistent with those obtained from the log-pulse XPCS results.

In all cases, the relaxation rates were slower than those obtained from the conventional XPCS measurements, indicating that the proposed method successfully avoided radiation-induced acceleration of the dynamics. In this experiment, the log-pulse XSVS results exhibited greater scatter than the log-pulse XPCS results. However, when the scattered intensity is extremely weak and the per-pixel count in a single frame is low, XPCS calculations become unreliable, whereas XSVS can provide more reliable dynamical information.

### 3.4. Discussion

**Figure 7:**
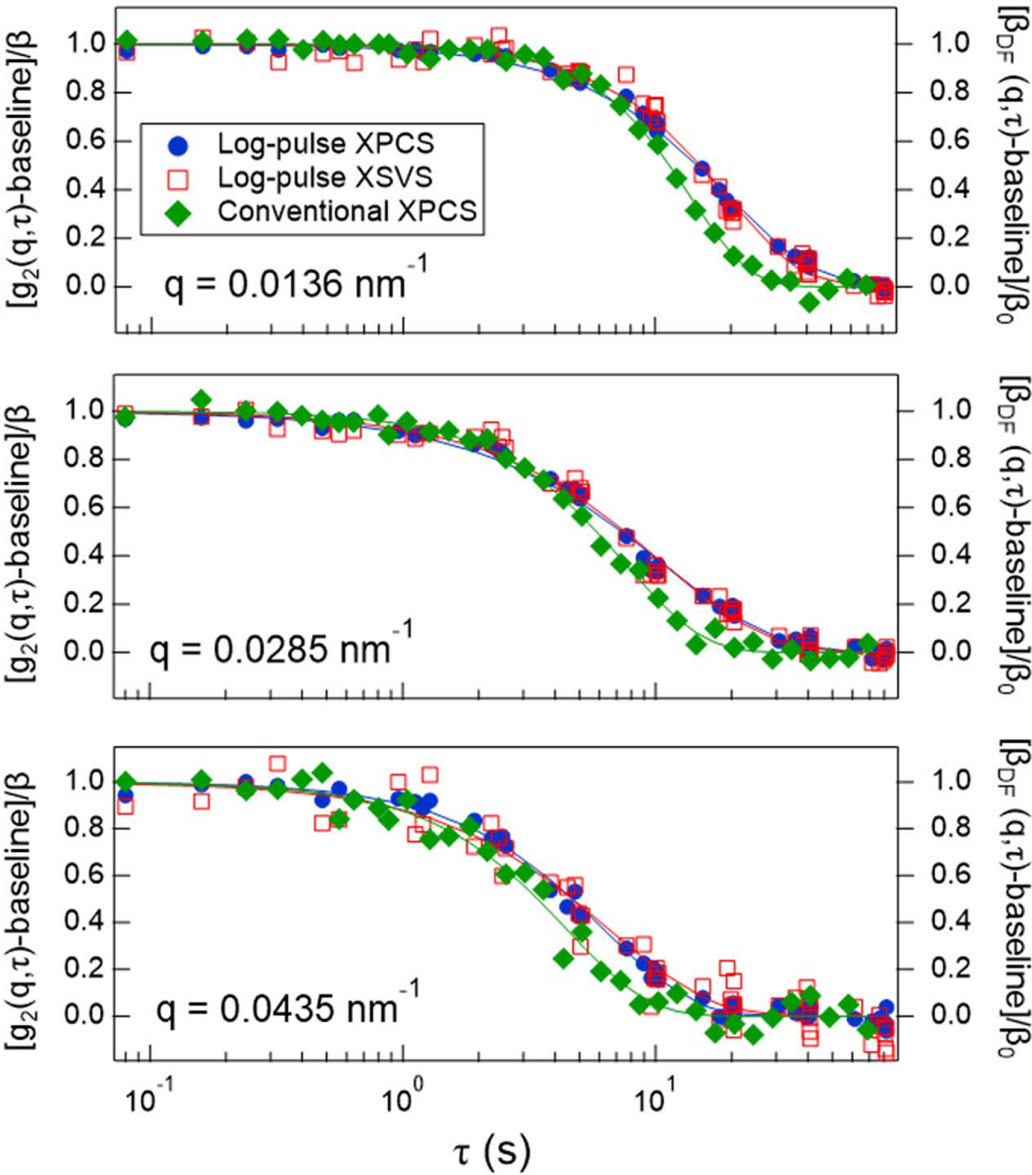
Three representative types of time correlation functions obtained from log-pulse XPCS, log-pulse XSVS and conventional XPCS, with the conventional XPCS data extracted from the two-time correlation function within the range *t* < 0.4 s.

Fig. 7 ▸ shows a comparison of the three types of time correlation functions corresponding to three different *q* values obtained from log-pulse XPCS, log-pulse XSVS and conventional XPCS, with the conventional XPCS data extracted from the two-time correlation function in the initial time range *t* < 0.4 s. For all datasets, the baseline was subtracted and the curves were normalized. The log-pulse XPCS and log-pulse XSVS data agreed well with each other, consistent with the results discussed above. In contrast, the correlation functions obtained from conventional XPCS showed slightly faster relaxation. However, the log-pulse results represent averages over 30 positions, while the conventional XPCS result shown here is based on a single position. Thus, it remains unclear whether the observed difference is significant. This will be examined in future work.

Finally, the measurements obtained are constrained by statistical limitations. For the current dataset, the error bars become larger at high *q*, primarily due to the reduced scattered intensity. At a high *q*, the signal-to-background ratio decreases, leading to a reduction in speckle contrast, as shown in Figs. 4 ▸(*a*) and 6 ▸(*a*). Consequently, it leads to larger uncertainties in the fitted relaxation parameters Γ and α. In addition to intensity-related effects, the limited number of frames in a single log-pulse sequence can inherently affect the estimation accuracy of Γ and α. Although the present approach could efficiently generate 55 distinct delay times from only 11 frames – significantly more than conventional XSVS schemes that provide a single delay time from two frames – the statistical quality remains lower than that of standard continuous XPCS measurements. As a result, the analysis is more susceptible to experimental noise and X-ray intensity fluctuations. These limitations can be mitigated in several ways. Combining datasets acquired using different frame-time patterns can improve the coverage of delay times and reduce gaps in the temporal sampling. Furthermore, increasing the number of measured positions enhances statistical reliability, but sample heterogeneity must be considered. The optimal number of positions depends on the degree of sample uniformity and therefore varies from system to system. Rather than fixing the number of measurement points in advance, it is more effective to monitor the convergence and scatter of the fitted parameters during the experiment and to determine the stopping criterion based on the observed statistical stability.

## 4. Conclusions

In this study, we performed non-uniform pulse XPCS measurements on a gel material that is highly sensitive to X-ray irradiation. In conventional XPCS measurements performed for comparison, time correlation functions were calculated from a 1000-frame time series and clear radiation-induced damage was observed. By contrast, in the non-uniform pulse XPCS measurements, only 11 speckle images were acquired, from which both the temporal autocorrelation function and relaxation behavior of the visibility contrast were evaluated. By reducing the total irradiation time to ∼100th of that used in conventional measurements, the dynamics could be assessed while effectively suppressing radiation damage. Moreover, the *q* dependence of the relaxation rates obtained from the two analysis methods (log-pulse XPCS and log-pulse XSVS) showed good agreement. The use of non-uniform pulses provides a sufficiently large number of delay-time combinations, enabling evaluation of deviations from a simple exponential form in the autocorrelation function. This capability is a significant advantage when investigating systems that exhibit non-simple Brownian motion.

Using the proposed method, we demonstrated that temporal autocorrelation functions can be obtained efficiently without requiring measurements at several positions. Although the method is limited to homogeneous samples in equilibrium, it is particularly effective when only a small amount of a uniform sample is available, as extensive spatial averaging is not necessary. As demonstrated in this experiment, this method is most effective for probing dynamics on millisecond and longer timescales. By enabling efficient and damage-suppressed XPCS measurements, this approach is expected to facilitate dynamical studies not only of polymer gels but also of a wide range of other systems, including organic materials and biological samples. As XPCS becomes accessible to a broader scientific community through upgraded light sources and improved instrumentation, radiation damage is expected to emerge as a central concern beyond the traditional XPCS user base. The proposed approach provides a practical and broadly applicable strategy for addressing this challenge.

## References

[bb1] Bandyopadhyay, R., Gittings, A. S., Suh, S. S., Dixon, P. K. & Durian, D. J. (2005). *Rev. Sci. Instrum.* **76**, 093110.

[bb2] Bikondoa, O. (2017). *J. Appl. Cryst.* **50**, 357–368. 10.1107/S1600576717000577 PMC5377338 28381968

[bb3] Brown, G., Rikvold, P. A., Sutton, M. & Grant, M. (1997). *Phys. Rev. E* **56**, 6601–6612.

[bb4] Carnis, J., Cha, W., Wingert, J., Kang, J., Jiang, Z., Song, S., Sikorski, M., Robert, A., Gutt, C., Chen, S. W., Dai, Y., Ma, Y., Guo, H., Lurio, L. B., Shpyrko, O., Narayanan, S., Cui, M., Kosif, I., Emrick, T., Russell, T. P., Lee, H. C., Yu, C. J., Grübel, G., Sinha, S. K. & Kim, H. (2014). *Sci. Rep.* **4**, 6017. 10.1038/srep06017 PMC4127496 25109363

[bb5] Chèvremont, W., Zinn, T. & Narayanan, T. (2024). *J. Synchrotron Rad.* **31**, 65–76. 10.1107/S1600577523008627 PMC10833426 37933847

[bb6] Chushkin, Y., Correa, J., Ignatenko, A., Pennicard, D., Lange, S., Fridman, S., Karl, S., Senfftleben, B., Lehmkühler, F., Westermeier, F., Graafsma, H. & Cammarata, M. (2025). *J. Synchrotron Rad.* **32**, 1220–1227. 10.1107/S1600577525006599 PMC12416413 40824695

[bb7] Chushkin, Y., Gulotta, A., Roosen-Runge, F., Pal, A., Stradner, A. & Schurtenberger, P. (2022). *Phys. Rev. Lett.* **129**, 238001. 10.1103/PhysRevLett.129.238001 36563210

[bb8] Dallari, F., Reiser, M., Lokteva, I., Jain, A., Möller, J., Scholz, M., Madsen, A., Grübel, G., Perakis, F. & Lehmkühler, F. (2021). *Appl. Sci.* **11**, 8037.

[bb9] DeCaro, C., Karunaratne, V. N., Bera, S., Lurio, L. B., Sandy, A. R., Narayanan, S., Sutton, M., Winans, J., Duffin, K., Lehuta, J. & Karonis, N. (2013). *J. Synchrotron Rad.* **20**, 332–338. 10.1107/S0909049512051825 23412491

[bb10] Dixon, P. K. & Durian, D. J. (2003). *Phys. Rev. Lett.* **90**, 184302. 10.1103/PhysRevLett.90.184302 12786009

[bb11] Frenzel, L., Lehmkühler, F., Lokteva, I., Narayanan, S., Sprung, M. & Grübel, G. (2019). *J. Phys. Chem. Lett.* **10**, 5231–5236. 10.1021/acs.jpclett.9b01690 31433650

[bb12] Girelli, A., Bin, M., Filianina, M., Dargasz, M., Anthuparambil, N. D., Möller, J., Zozulya, A., Andronis, I., Timmermann, S., Berkowicz, S., Retzbach, S., Reiser, M., Raza, A. M., Kowalski, M., Sayed Akhundzadeh, M., Schrage, J., Woo, C. H., Senft, M. D., Reichart, L. F., Leonau, A., Prince, P. R., Chèvremont, W., Seydel, T., Hallmann, J., Rodriguez-Fernandez, A., Pudell, J. E., Brausse, F., Boesenberg, U., Wrigley, J., Youssef, M., Lu, W., Jo, W., Shayduk, R., Guest, T., Madsen, A., Lehmkühler, F., Paulus, M., Zhang, F., Schreiber, F., Gutt, C. & Perakis, F. (2025). *Nat. Commun.* **16**, 10814. 10.1038/s41467-025-66972-6 PMC12669764 41318598

[bb13] Goodman, J. W. (1985). *Statistical optics.* New York: Wiley.

[bb14] Gutt, C., Stadler, L. M., Duri, A., Autenrieth, T., Leupold, O., Chushkin, Y. & Grübel, G. (2009). *Opt. Express* **17**, 55–61. 10.1364/oe.17.000055 19129872

[bb15] Hernández, R., Criado, M., Nogales, A., Sprung, M., Mijangos, C. & Ezquerra, T. A. (2015). *Macromolecules* **48**, 393–399.

[bb16] Hoshino, T., Murakami, D., Tanaka, Y., Takata, M., Jinnai, H. & Takahara, A. (2013). *Phys. Rev. E* **88**, 032602. 10.1103/PhysRevE.88.032602 24125287

[bb17] Hoshino, T., Okamoto, Y., Yamamoto, A. & Masunaga, H. (2021). *Sci. Rep.* **11**, 9767. 10.1038/s41598-021-89155-x PMC8129072 34001939

[bb18] Hruszkewycz, S. O., Sutton, M., Fuoss, P. H., Adams, B., Rosenkranz, S., Ludwig, K. F. Jr, Roseker, W., Fritz, D., Cammarata, M., Zhu, D., Lee, S., Lemke, H., Gutt, C., Robert, A., Grübel, G. & Stephenson, G. B. (2012). *Phys. Rev. Lett.* **109**, 185502. 10.1103/PhysRevLett.109.185502 23215295

[bb19] Inoue, I., Shinohara, Y., Watanabe, A. & Amemiya, Y. (2012). *Opt. Express* **20**, 26878–26887. 10.1364/OE.20.026878 23187541

[bb20] Ishiguro, N., Kaneko, F., Abe, M., Takayama, Y., Yoshida, J., Hoshino, T., Takazawa, S., Uematsu, H., Sasaki, Y., Okawa, N., Takahashi, K., Takizawa, H., Kishimoto, H. & Takahashi, Y. (2024). *Appl. Phys. Expr.* **17**, 052006.

[bb21] Jo, W., Möller, J., Hallmann, J., Wrigley, J., Pudell, J.-E., Boesenberg, U., Brausse, F., Rodriguez-Fernandez, A., Zozulya, A., Shayduk, R. & Madsen, A. (2025). *J. Synchrotron Rad.* **32**, 669–677. 10.1107/S1600577525002875 PMC12067325 40278848

[bb22] Jo, W., Westermeier, F., Rysov, R., Leupold, O., Schulz, F., Tober, S., Markmann, V., Sprung, M., Ricci, A., Laurus, T., Aschkan, A., Klyuev, A., Trunk, U., Graafsma, H., Grübel, G. & Roseker, W. (2021). *IUCrJ* **8**, 124–130. 10.1107/S2052252520015778 PMC7792991 33520248

[bb23] Lehmkühler, F., Dallari, F., Jain, A., Sikorski, M., Möller, J., Frenzel, L., Lokteva, I., Mills, G., Walther, M., Sinn, H., Schulz, F., Dartsch, M., Markmann, V., Bean, R., Kim, Y., Vagovic, P., Madsen, A., Mancuso, A. P. & Grübel, G. (2020). *Proc. Natl Acad. Sci. USA* **117**, 24110–24116. 10.1073/pnas.2003337117 PMC7533660 32934145

[bb24] Lehmkühler, F., Kwaśniewski, P., Roseker, W., Fischer, B., Schroer, M. A., Tono, K., Katayama, T., Sprung, M., Sikorski, M., Song, S., Glownia, J., Chollet, M., Nelson, S., Robert, A., Gutt, C., Yabashi, M., Ishikawa, T. & Grübel, G. (2015). *Sci. Rep.* **5**, 17193. 10.1038/srep17193 PMC4661692 26610328

[bb25] Lehmkühler, F., Valerio, J., Sheyfer, D., Roseker, W., Schroer, M. A., Fischer, B., Tono, K., Yabashi, M., Ishikawa, T. & Grübel, G. (2018). *IUCrJ* **5**, 801–807. 10.1107/S2052252518013696 PMC6211528 30443363

[bb26] Lhermitte, J. R. M., Rogers, M. C., Manet, S. & Sutton, M. (2017). *Rev. Sci. Instrum.* **88**, 015112. 10.1063/1.4974099 28147652

[bb27] Li, L., Kwaśniewski, P., Orsi, D., Wiegart, L., Cristofolini, L., Caronna, C. & Fluerasu, A. (2014). *J. Synchrotron Rad.* **21**, 1288–1295. 10.1107/S1600577514015847 25343797

[bb99] Majumdar, A., Li, H., Muhunthan, P., Späh, A., Song, S., Sun, Y., Chollet, M., Sokaras, D., Zhu, D. & Ihme, M. (2024). *Nat Commun*, **15**, 10540. 10.1038/s41467-024-54782-1 PMC11615208 39627208

[bb28] Malik, A., Sandy, A. R., Lurio, L. B., Stephenson, G. B., Mochrie, S. G. J., McNulty, I. & Sutton, M. (1998). *Phys. Rev. Lett.* **81**, 5832–5835.

[bb29] Mandel, L. (1959). *Proc. Phys. Soc.* **74**, 233–243.

[bb30] Möller, J., Reiser, M., Hallmann, J., Boesenberg, U., Zozulya, A., Rahmann, H., Becker, A.-L., Westermeier, F., Zinn, T., Sprung, M., Narayanan, T., Gutt, C. & Madsen, A. (2021). *New J. Phys.* **23**, 093041.

[bb31] Nishimori, N., Asaka, T., Ueshima, K., Hosaka, Y., Obara, S. & Kan, K. (2025). *J. Phys. Conf. Ser.* **3010**, 012011.

[bb98] Obara, S., Ueshima, K., Asaka, T., Hosaka, Y., Kan, K., Nishimori, N., Aoki, T., Asano, H., Haga, K., *Iba*, Y., Ihara, A., Ito, K., Iwashita, T., Kadowaki, M., Kanahama, R., Kobayashi, H., Kobayashi, H., Nishihara, H., Nishikawa, M., Oikawa, H., Saida, R., Sakuraba, K., Sugimoto, K., Suzuki, M., Takahashi, K., Takahashi, S., Tanaka, T., Tsuchiyama, T., Yoshioka, R., Aoki, T., Dewa, H., Fujita, T., Kawase, M., Kiyomichi, A., Hamano, T., Masaki, M., Masuda, T., Matsubara, S., Okada, K., Saji, C., Taniuchi, T., Taniuchi, Y., Ueda, Y., Yamaguchi, H., Yanagida, K., Fukami, K., Hosoda, N., Ishii, M., Itoga, T., Iwai, E., Magome, T., Oishi, M., Ohshima, T., Kondo, C., Sakurai, T., Shoji, M., Sugimoto, T., Takano, S., Tamura, K., Watanabe, T., Tomai, T., Azumi, N., Inagaki, T., Maesaka, H., Takahashi, S., Tanaka, T., Inoue, S., Kumazawa, H., Moriya, K., Sakai, K., Seno, T., Sumitomo, H., Takesako, R., Tanaka, S., Yamamoto, R., Yokomachi, K., Yoshioka, M., Hara, T., Matsui, S., Hiraiwa, T., Tanaka, H. & Ego, H. (2025). *Phys. Rev. Accel. Beams* **28**, 020701.

[bb32] Reiser, M., Girelli, A., Ragulskaya, A., Das, S., Berkowicz, S., Bin, M., Ladd-Parada, M., Filianina, M., Poggemann, H. F., Begam, N., Akhundzadeh, M. S., Timmermann, S., Randolph, L., Chushkin, Y., Seydel, T., Boesenberg, U., Hallmann, J., Möller, J., Rodriguez-Fernandez, A., Rosca, R., Schaffer, R., Scholz, M., Shayduk, R., Zozulya, A., Madsen, A., Schreiber, F., Zhang, F., Perakis, F. & Gutt, C. (2022). *Nat. Commun.* **13**, 5528. 10.1038/s41467-022-33154-7 PMC9490738 36130930

[bb33] Roseker, W., Hruszkewycz, S. O., Lehmkühler, F., Walther, M., Schulte-Schrepping, H., Lee, S., Osaka, T., Strüder, L., Hartmann, R., Sikorski, M., Song, S., Robert, A., Fuoss, P. H., Sutton, M., Stephenson, G. B. & Grübel, G. (2018). *Nat. Commun.* **9**, 1704. 10.1038/s41467-018-04178-9 PMC5923200 29703980

[bb34] Ruta, B., Czakkel, O., Chushkin, Y., Pignon, F., Nervo, R., Zontone, F. & Rinaudo, M. (2014). *Soft Matter* **10**, 4547–4554. 10.1039/c4sm00704b 24817660

[bb35] Ruta, B., Zontone, F., Chushkin, Y., Baldi, G., Pintori, G., Monaco, G., Rufflé, B. & Kob, W. (2017). *Sci. Rep.* **7**, 3962. 10.1038/s41598-017-04271-x PMC5479813 28638053

[bb36] Shinohara, Y., Osaka, T., Inoue, I., Iwashita, T., Dmowski, W., Ryu, C. W., Sarathchandran, Y. & Egami, T. (2020). *Nat. Commun.* **11**, 6213. 10.1038/s41467-020-20036-z PMC7718898 33277499

[bb37] Sun, Y., Carini, G., Chollet, M., Decker, F. J., Dunne, M., Fuoss, P., Hruszkewycz, S. O., Lane, T. J., Nakahara, K., Nelson, S., Robert, A., Sato, T., Song, S., Stephenson, G. B., Sutton, M., Van Driel, T. B., Weninger, C. & Zhu, D. (2021). *Phys. Rev. Lett.* **127**, 058001. 10.1103/PhysRevLett.127.058001 34397240

[bb38] Sun, Y., Montana-Lopez, J., Fuoss, P., Sutton, M. & Zhu, D. (2020). *J. Synchrotron Rad.* **27**, 999–1007. 10.1107/S1600577520006773 PMC7336177 33566009

[bb39] Sutton, M. (2008). *C. R. Phys.* **9**, 657–667.

[bb40] Timmermann, S., Anthuparambil, N. D., Girelli, A., Begam, N., Kowalski, M., Retzbach, S., Senft, M. D., Akhundzadeh, M. S., Poggemann, H. F., Moron, M., Hiremath, A., Gutmüller, D., Dargasz, M., Öztürk, O., Paulus, M., Westermeier, F., Sprung, M., Ragulskaya, A., Zhang, F., Schreiber, F. & Gutt, C. (2023). *Sci. Rep.* **13**, 11048. 10.1038/s41598-023-38059-z PMC10329714 37422480

[bb41] Verwohlt, J., Reiser, M., Randolph, L., Matic, A., Medina, L. A., Madsen, A., Sprung, M., Zozulya, A. & Gutt, C. (2018). *Phys. Rev. Lett.* **120**, 168001. 10.1103/PhysRevLett.120.168001 29756927

[bb42] Yavitt, B. M., Salatto, D., Zhou, Y., Huang, Z., Endoh, M., Wiegart, L., Bocharova, V., Ribbe, A. E., Sokolov, A. P., Schweizer, K. S. & Koga, T. (2021). *ACS Nano* **15**, 11501–11513. 10.1021/acsnano.1c01283 34128655

[bb43] Zhang, Q., Dufresne, E. M., Narayanan, S., Maj, P., Koziol, A., Szczygiel, R., Grybos, P., Sutton, M. & Sandy, A. R. (2018). *J. Synchrotron Rad.* **25**, 1408–1416. 10.1107/S1600577518009074 30179180

